# Detection of Acute Pulmonary Embolism by Bedside Ultrasound in a Patient Presenting in PEA Arrest: A Case Report

**DOI:** 10.1155/2012/794019

**Published:** 2012-05-31

**Authors:** Hangyul Chung-Esaki, Roneesha Knight, Jeanne Noble, Ralph Wang, Zlatan Coralic

**Affiliations:** Department of Emergency Medicine, University of California San Francisco, Room M24, 505 Parnassus Avenue, San Francisco, CA 94143, USA

## Abstract

Optimal management of the critically ill patient in shock requires rapid identification of its etiology. We describe a successful application of an emergency physician performed bedside ultrasound in a patient presenting with shock and subsequent cardiac arrest. Pulmonary embolus was diagnosed using bedside echocardiogram and confirmed with CTA of the thorax. Further validation and real-time implementation of this low-cost modality could facilitate the decision to implement thrombolytics for unstable patients with massive pulmonary embolism who cannot undergo formal radiographic evaluation.

## 1. Introduction

Identifying the possible causes of shock in the critically ill patient in the emergency department (ED) is challenging due to limited information, broad differentials, and the severity of illness demanding rapid intervention. A quick narrowing of one's differential diagnosis using bedside ultrasound may tailor therapy and guide further workup, especially in patients deemed too unstable to undergo computed tomography imaging. In these situations, ultrasound may be invaluable as a low-cost, real-time, noninvasive modality which allows serial bedside examinations, possibly identifying the cause of hypotension or cardiac arrest. We discuss a case of massive pulmonary embolism diagnosed by bedside ultrasound.

## 2. Case Presentation

A 53-year-old man was brought in by ambulance with the chief complaint of multiple “fainting” episodes. He was reported by the paramedics to be hypotensive and tachycardic prior to arrival. His past medical history was significant for hypertension, chronic renal insufficiency, and gouty arthritis. On presentation to the emergency department, the patient appeared critically ill with generalized pallor, perioral cyanosis, with a heart rate of 133 beats per minute (bpm), blood pressure of 130/106 mm Hg, and oxygen saturation of 100% on 15 L O_2_ via a nonrebreather mask. His exam was significant for grunting, otherwise clear bilateral breath sounds, rapid but regular heart tones, weak femoral pulses, and symmetric, non-edematous lower extremities.

**Figure 1:**
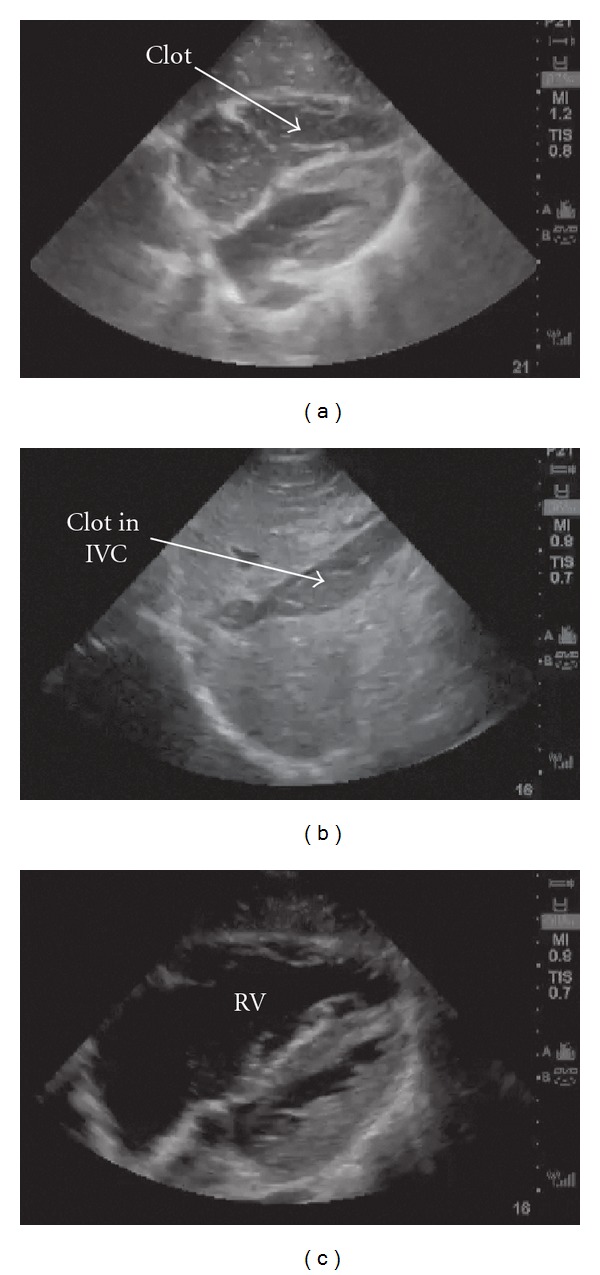
Initial bedside ultrasound demonstrated thrombus in the RV (a) and the IVC (b). A repeat ultrasound demonstrated acute RV enlargement (c).

**Figure 2:**
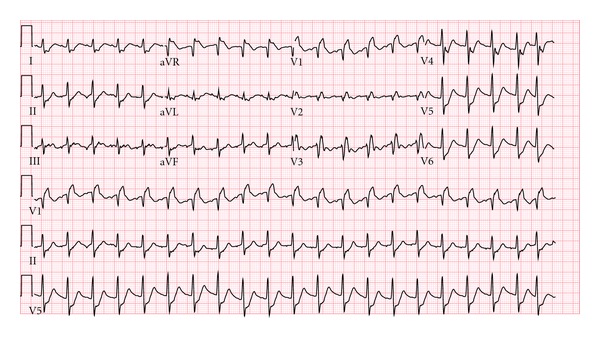
EKG demonstrates new right bundle branch block.

**Figure 3:**
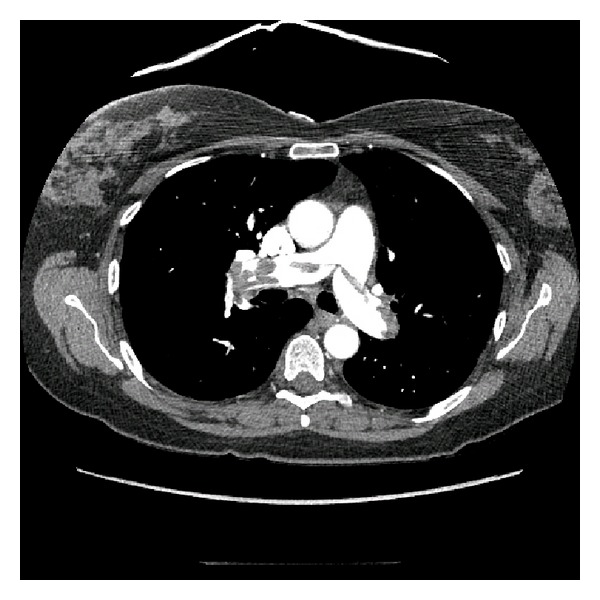
CT angiogram of the chest demonstrating bilateral pulmonary embolus.

Shortly after arrival, a bedside ultrasound was performed, demonstrating a thrombus in the right ventricle (RV) and inferior vena cava (IVC) (Figures 1(a) and 1(b)). The patient lost cardiac motion during the ultrasound, and cardiopulmonary resuscitation (CPR) was initiated with return of spontaneous circulation within one minute. He was emergently intubated, and his postintubation oxygen saturation was noted to be 60%, despite 100% FiO_2_ and confirmation of adequate tube placement. A repeat bedside ultrasound was performed with visualization of an enlarged right ventricle (Figure 1(c)), and an EKG demonstrated a new right bundle branch block (Figure 2), highly suspicious for a massive pulmonary embolus. Shortly thereafter, the patient again lost pulses requiring CPR and 1 mg IV epinephrine, with subsequent return of spontaneous circulation in three minutes. The patient's blood pressure was maintained on a continuous infusion of epinephrine, and bolus dosing of alteplase (tPA) was being prepared while a CT angiogram (CTA) of the thorax was ordered. The CTA confirmed the diagnosis of bilateral massive pulmonary emboli (PE) (Figure 3), and alteplase was administered at a bolus dose of 0.6 mg/kg over 2 minutes followed by a heparin infusion and admitted to the ICU without further hemodynamic decompensation.

**Figure 4:**
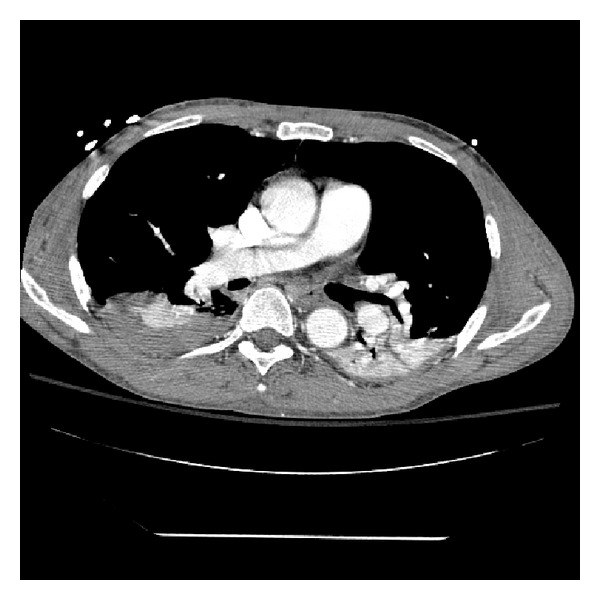
CT angiogram of the chest after thrombolysis and anticoagulation with resolution of visible thrombi.

The patient's course was complicated by bilateral pneumothoraces, acute renal failure, and subconjunctival hemorrhage. However, he was ultimately extubated and discharged home with full neurologic recovery, and a repeat CTA chest demonstrated resolution of pulmonary thrombi (Figure 4). A deep venous thrombosis (DVT) in the left distal popliteal vein with an aneurismal dilation of the popliteal vein was diagnosed during his inpatient stay, thought to be related a recent flare of gouty arthritis. The patient is currently awaiting outpatient work-up for possible coagulopathy and continues on warfarin anticoagulation.

## 3. Discussion

This case demonstrates successful application of bedside ultrasound in the diagnosis of massive PE in a patient resulting in PEA arrest. Although thrombolytics were given immediately after confirmation of PE by thoracic CTA, the sonographic evidence of a right sided thrombus (Figure 1(a)) and an enlarged IVC (Figure 1(b)) with acute RV dilation (Figure 1(c)) strongly suggested the diagnosis. In fact, the alteplase bolus was prepared as the patient was transported to the CT scanner, with a plan to administer thrombolytics emergently in the case of repeat arrest or upon confirmation of massive PE.

Hesitancy to initiate a potentially harmful therapy such as thrombolytics is understandable, given the overall poor sensitivity and specificity of ED-performed sonography for PE. Sonographic findings which support the diagnosis of acute PE include direct signs such as free-floating thrombus in the right heart or pulmonary artery or indirect signs such as RV dilation (>1 : 1 RV/LV ratio), RV systolic dysfunction, flattening or bowing of the intraventricular septum into the LV, IVC dilation without inspiratory collapse, or evidence of DVT on compression ultrasound of the lower extremities [1, 2]. Despite their moderate to high specificity, these signs overall have poor sensitivity—RV dilation and dysfunction have sensitivities of 29% and 51%, respectively, with a combined sensitivity of 52–56% [1], and only 30–40% of patients with acute PE may demonstrate an abnormal finding on echocardiogram [2]. Also, while RV enlargement and RV dysfunction may portend a poor prognosis in acute PE (OR for mortality 2.53, 95% CI 1.17–5.50) [3], these findings alone without a consistent clinical picture are not specific and may also be seen in COPD, obstructive sleep apnea, pulmonary hypertension, and right sided myocardial infarction. Perhaps the most sensitive and specific indirect sign is the McConnell sign, or hypokinesis of the RV mid-free wall with preserved apical contractility as seen in the four-chamber view. Originally described as 77% sensitive and 94% specific, a much lower specificity of 33% was found in a recent study which included patients with RV infarction [4]. These reported sensitivities and specificity are based on formal comprehensive transthoracic echocardiography, and ED-performed sonography may be even less sensitive and would not be adequate to rule out PE. However, according to the joint policy published by the American Society of Echocardiography (ASE) and the American College of Emergency Physicians (ACEP), in the hemodynamically *unstable *patient or a patient in cardiac arrest who carries a high probability of pulmonary embolism, visualizing these signs could guide further testing or initiation of therapy such as thrombolytic [1].

In this case, the direct visualization of a clot in the IVC and right atrium, with acute RV enlargement coinciding with clot disappearance, strongly suggested a PE contributing to the patient's hemodynamic instability. The presence of clot on echocardiogram or “thrombosis in transit” has been described previously [5–13], including in emergency department settings [14–16], and is thought to be relatively rare, estimated to be present in 4–18% of acute PE [12, 15]. Clots are often described as free, ovoid, or coiled densities rotating within the atrium [17, 18], attached to the atrial or ventricular wall or septum, or trapped in the tricuspid valve [19], chordae tendineae, or right ventricular papillary muscles [9]. These clots are thought to cause microemboli or massive pulmonary embolism [18] as in our case. In situ movement of the clot has also been described in various case studies with concomitant presence of RV dilatation [8, 12], but our case is the first case which visualized a clot within the RV and IVC, documented its subsequent disappearance and coincident RV dilatation, consistent with pulmonary embolism.

Visualization of right heart thrombus may also suggest poor response to anticoagulation alone based on a small case series [7] and other case reports [9]. Although treatment of right heart thrombi remains controversial, two larger retrospective reviews suggest that these patients may require aggressive treatment such as thrombolysis or thrombectomy. Torbicki et al. evaluated the prognostic significance of right heart thrombi within 2,454 cases of PE from the International Cooperative Pulmonary Embolism Registry and concluded that patients with right heart thrombi had higher rates of hemodynamic instability and/or mortality. They suggested that anticoagulation alone may not be adequate treatment for such patients [13]. Similarly, Rose et al. evaluated the largest sample of patients with right heart thromboembolism to date and suggested that thrombolytic therapy was associated with decreased mortality (11.3%) when compared to anticoagulation (28.6%) or surgical therapy (23.8%) [5].

Through visualization of right heart thrombus, RV dilatation, and acute cor pulmonale, use of bedside ultrasound can help guide diagnosis and therapy in pulmonary embolism and add prognostic information. Similarly, the use of bedside ultrasound in the crashing patient with cardiac arrest may help distinguish between asystole, pulseless electrical activity, and pseudopulseless electrical activity, while identifying alternative diagnoses (pericardial effusion, hypovolemia, and cardiac dysfunction) and guiding emergency procedures [20]. In fact, just as the focused assessment using sonography in trauma (FAST) has become a standard component of Advanced Trauma Life Support (ATLS), selective use of ultrasound in patients with shock provides a real-time systematic evaluation of the undifferentiated patient with hypotension. Recently, the RUSH protocol, which includes the evaluation of “the pump,” “the tank,” and “the pipes” [21], has been proposed as a formal paradigm in the evaluation of shock. While each element may be selectively used based on the clinical scenario, such paradigms provide a systematic framework for evaluating critically ill patients. Early “goal-directed use of ultrasound” in nontrauma patients with undifferentiated hypotension has the potential to improve patient outcome by decreasing time to diagnosis and appropriate therapy.

Lastly, the thrombolytic dosage used in this case (0.6 mg/kg of alteplase as an IV bolus) is a smaller but faster bolus than the current FDA and AHA recommendations for pulmonary embolism (100 mg alteplase IV over 2 hours) [3]. However, the original FDA recommendations were based on a randomized study of 45 patients comparing the efficacy of alteplase to urokinase [22]. Additional studies have explored alternative regimens including a 0.6 mg/kg IV bolus regimen [23, 24] and concluded there were no overall differences in symptoms or outcome. A more recent study suggested equivalence in reduced bolus dose of alteplase (50 mg versus 100 mg IV) [25]. However, two-thirds of the patients had a large clot burden without hemodynamic compromise, and majority of patients had a BMI <30, limiting the relevancy of their findings to submassive PE cases and nonobese patients. Optimal agent and dosing of thrombolytics remain controversial, and current studies evaluating tenecteplase, a new recombinant thrombolytic given as a bolus dose with higher fibrin specificity [26], may further alter our current management of PE. In addition, the most recent AHA guidelines recommend considering thrombolysis in submassive PE with significant RV dysfunction as evidenced by biomarkers or RV dilatation on echocardiogram, which not only may increase our use of thrombolytics, but may also serve to encourage the use of bedside echocardiogram in the ED.

## 4. Conclusion

Bedside ultrasound may help differentiate between the etiologies of hypotension in the unstable patient. In cases of acute PE, evidence of right heart thrombus on real-time ultrasound portends a poor prognosis, and these patients may benefit from more aggressive treatment such as thrombectomy or thrombolysis as in this case. The optimal dosing of thrombolytics for acute PE is controversial and is in need of further investigation.
